# Unsupervised Few-Shot Feature Learning via Self-Supervised Training

**DOI:** 10.3389/fncom.2020.00083

**Published:** 2020-10-14

**Authors:** Zilong Ji, Xiaolong Zou, Tiejun Huang, Si Wu

**Affiliations:** ^1^State Key Laboratory of Cognitive Neuroscience & Learning, Beijing Normal University, Beijing, China; ^2^School of Electronics Engineering & Computer Science, Peking University, Beijing, China; ^3^IDG/McGovern Institute for Brain Research, PKU-Tsinghua Center for Life Sciences, Peking University, Beijing, China

**Keywords:** unsupervised, few-shot learning, clustering, pseudo labels, episodic learning

## Abstract

Learning from limited exemplars (few-shot learning) is a fundamental, unsolved problem that has been laboriously explored in the machine learning community. However, current few-shot learners are mostly supervised and rely heavily on a large amount of labeled examples. Unsupervised learning is a more natural procedure for cognitive mammals and has produced promising results in many machine learning tasks. In this paper, we propose an unsupervised feature learning method for few-shot learning. The proposed model consists of two alternate processes, progressive clustering and episodic training. The former generates pseudo-labeled training examples for constructing episodic tasks; and the later trains the few-shot learner using the generated episodic tasks which further optimizes the feature representations of data. The two processes facilitate each other, and eventually produce a high quality few-shot learner. In our experiments, our model achieves good generalization performance in a variety of downstream few-shot learning tasks on Omniglot and MiniImageNet. We also construct a new few-shot person re-identification dataset FS-Market1501 to demonstrate the feasibility of our model to a real-world application.

## 1. Introduction

Few-shot learning, which aims to accomplish a learning task by using very few training examples, is receiving increasing attention in both of the machine learning and cognitive science community. The challenge of few-shot learning lies on the fact that traditional techniques such as fine-tuning would normally incur overfitting (Wang et al., 2018). To overcome this, an episodic training paradigm was proposed (Vinyals et al., 2016). In such a paradigm, episodic training replaces the conventional mini-batch training, such that a batch of episodic tasks, each of which have the same setting as the testing environment, are presented to the learning model; and in each episodic task, the model learns to predict the classes of unlabeled points (the query set) using very few labeled examples (the support set). By this, the learning model acquires the transferable knowledge across tasks, and due to the consistency between the training and testing environments, the model is able to generalize to novel but related downstream tasks. Although this set-to-set few-shot learning paradigm has made great progress, in its current supervised form, it requires a large number of labeled examples for constructing episodic tasks, which is often infeasible or too expensive in practice. So, can we build up a few-shot learner in the paradigm of episodic training using only unlabeled data?

It is well-known that humans have the remarkable ability to learn a concept when given only several exposures to its instances, for example, young children can effortlessly learn and generalize the concept of “giraffe” after seeing a few pictures of giraffes. While the specifics of the human learning process are complex (trial-based, perpetual, multi-sourced, and simultaneous for multiple tasks) and yet to be solved, previous works agree that its nature is progressive and unsupervised in many cases (Dupoux, 2018). Given a set of unlabeled items, humans are able to organize them into different clusters by comparing one with another. The comparing or associating process follows a *coarse-to-fine* manner. At the beginning of learning, humans tend to group items based on fuzzy-rough knowledge such as color, shape, or size. Subsequently, humans build up associations between items using more fine-grained knowledge, i.e., stripes of images, functions of items, or other domain knowledge. Furthermore, humans can extract representative representations across categories and apply this capability to learn new concepts (Kemp et al., 2010; Wang et al., 2014; Gopnik and Bonawitz, 2015).

**Figure 1:**
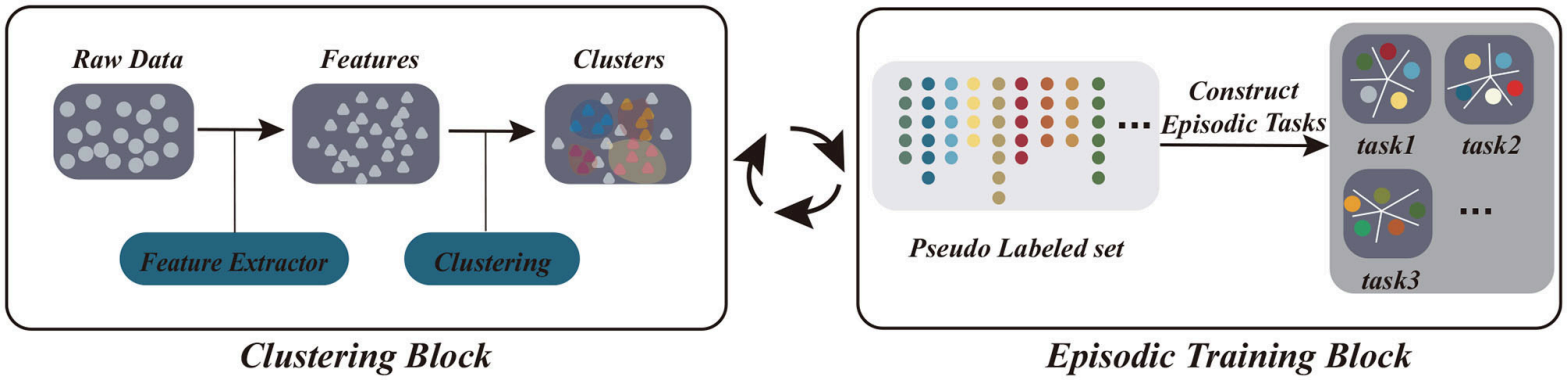
The scheme of our model UFLST, which integrates two iterative processes: clustering and episodic training. At each iteration, unlabeled datapoints are clustered based on the extracted features, and pseudo labels are assigned according to the cluster identities. After clustering, a set of episodic tasks are constructed by sampling from the pseudo labeled data, and the few-shot learner is trained, which further optimizes feature representations. The two processes are repeated.

In the present study, inspired by the unsupervised and progressive characteristics of human learning, we propose an unsupervised model for few-shot learning via a self-supervised training procedure (UFLST). Different from previous unsupervised learning methods, our model integrates unsupervised learning and episodic training into a unified framework, which facilitates feature extraction and model training iteratively. Basically, we adopt the episodic training paradigm, taking advantage of its capability of extracting transferable knowledge across tasks, but we use an unsupervised strategy to construct episodic tasks. Specifically, we apply progressive clustering to generate pseudo labels for unlabeled data, and this is done alternatively with feature optimization via few-shot learning in an iterative manner (Figure 1). Initially, unlabeled data points are assigned into several clusters, and we sample a few training examples from each cluster together with their pseudo labels (the identities of clusters) to construct a set of episodic tasks having the same setting as the testing environment. We then train the few-shot learner using the constructed episodic tasks and obtain improved feature representations for the data. In the next round, we use the improved features to re-cluster data points, generating new pseudo labels and constructing new episodic tasks, and train the few-shot learner again. The above two steps are repeated till a stopping criterion is reached. After training, we expect that the few-shot learner has acquired the transferable knowledge (the optimized feature representations) suitable for a novel task of the same setting as in the episodic training. Using benchmark datasets, we demonstrate that our model outperforms other unsupervised few-shot learning methods and approaches to the performances of fully supervised models.

### 1.1. Related Works

In the paradigm of episodic training, few-shot learning algorithms can be divided into two main categories: “learning to optimize” and “learning to compare.” The former aims to develop a learning algorithm which can adapt to a new task efficiently using only few labeled examples or with only few steps of parameter updating (Andrychowicz et al., 2016; Ravi and Larochelle, 2016; Finn et al., 2017; Mishra et al., 2017; Nichol and Schulman, 2018; Rusu et al., 2018), and the latter aims to learn a proper embedding function, so that prediction is based on the distance (metric) of a novel example to the labeled instances (Vinyals et al., 2016; Snell et al., 2017; Liu et al., 2018; Ren et al., 2018; Sung et al., 2018). In the present study, we focus on the “learning to compare” framework, although methods belonging to the other framework can also be integrated into our model.

A number of unsupervised few-shot learning models have been developed recently. Hsu et al. (2018) proposed a method called CACTUs, which constructs tasks from unlabeled data by partitioning features extracted by some prior unsupervised feature learning methods, e.g., ACAI, BiGAN, and DeepCluster in an automatic way and performs meta-learning over the constructed tasks. Khodadadeh et al. (2018) proposed a method called UMTRA, which utilizes the statistical diversity properties and domain-specific augmentations to generate training and validation data. Antoniou and Storkey (2019) proposed a similar model called AAL, which uses data augmentations of the unlabeled support set to generate the query data. All these methods construct episodic tasks with the aid of unsupervised feature embedding or data augmentation; whereas in our method, the construction of episodic tasks and model training are performed iteratively within the same few-shot embedding network, and they facilitate each other.

The idea of iterative training used in our model is a type of self-supervised training, which aims to artificially generate pseudo labels for unlabeled data and then perform feature learning as in the supervised manner iteratively. It is quite useful when supervisory signals are not available or too expensive (de Sa, 1994). This idea was first applied in NLP tasks, which aims to self-train a two-phase parser-reranker system using unlabeled data (McClosky et al., 2006). Xie et al. (2016) proposed a Deep Embedded Clustering network to jointly learn cluster centers and network parameters. Caron et al. (2018) further proposed strategies to solve the degenerated solution problem during deep clustering. Fan et al. (2018) and Song et al. (2018) applied the iterative training idea to the person re-identification task, both of which aim to transfer the extracted feature representations to an unseen domain. However, none of these studies have considered integrating iterative clustering and episodic training in unsupervised few-shot learning as we do in this work.

## 2. Materials and Methods

### 2.1. Preliminaries

In this section, we introduce the proposed model UFLST in detail. Consider a M-way K-shot classification task. Our goal is to train a few-shot learner based on the unlabeled data set $X={\left\{{\text{x}}_{i}\right\}}_{i=1}^{N}$, where *N* is the total number of unlabeled datapoints. The previous studies have demonstrated that by matching the training and testing paradigms, episodic learning can extract transferable knowledge across tasks suitable for few-shot classification (Vinyals et al., 2016). In the supervised setting, one can easily construct a set of episodic tasks, with each task having *K* training examples {(**x**_*k*_, *y*_*k*_)} per class to learn the few-shot classifier and *Q* query examples per class to evaluate the learned classifier. Totally, there are *K* + *Q* examples for each of *M* classes in each episodic task. In the unsupervised setting, however, we do not have labeled data to construct episodic tasks directly. Therefore, we consider using pseudo labels generated by a clustering algorithm to support episodic learning. Different from the previous work (Hsu et al., 2018) which uses a prior trained feature embedding network to extract fixed representations of data, data representations in our model are dynamically fine-tuned along with the episodic training.

Let us denote the embedding function in UFLST as *f*_θ_, which takes X as the input and outputs the corresponding feature vector $Z=\left\{{\text{z}}_{i}\right\}$, for *i* = 1, …, *N*, where θ represents the network parameters. Firstly, we cluster the unlabeled data based on the embedding features Z and obtain the pseudo labels of data {*y*_*i*_}, for *i* = 1, …, *N*. Secondly, using the pseudo labeled data, we construct a set of episodic tasks $T=\left\{T_{1},T_{2},...,T_{S}\right\}$, with *S* the number of constructed tasks in the current iteration, and carry out episodic learning, which improves the embedding features Z further. Notably, each episodic task *T*_*s*_ has the same setting as the application, i.e., it is a M-way K-shot classification. The above two steps are performed iteratively until a stopping criterion is reached. Below describes the two training processes in more detail.

### 2.2. Data Clustering

#### 2.2.1. Distance Metric for Clustering

To cluster data, the first is to choose a suitable metric measuring the distance between data points. For constructing a large number of episodic tasks, an over-complete partition of data points is preferred, leading to a large number of classes with a small number of examples in each class. In such a situation, the conventional Euclidean distance or the Cosine distance is no longer optimal. Inspired by the re-ranking idea used in object retrieval as a post-processing tool to improve the retrieval accuracy, we propose to use the k-reciprocal Jaccard distance (KRJD) metric (Qin et al., 2011; Zhong et al., 2017) as the distance measurement between two feature points **z**_*i*_ and **z**_*j*_, which is written as

(1) $$\begin{array}{l} J_{ij}=1-\frac{\left|R\left({\text{z}}_{i},k\right)\cap R\left({\text{z}}_{j},k\right)\right|}{\left|R\left({\text{z}}_{i},k\right)\cup R\left({\text{z}}_{j},k\right)\right|}. \end{array}$$

Here, *R*(**z**, *k*) counts the k-reciprocal nearest neighbors of a feature point **z** and is given by

(2) $$\begin{array}{l} R\left(\text{z},k\right)=\left\{{\text{z}}_{j}\;|\left({\text{z}}_{j}\in N\left(\text{z},k\right)\right)\cap \left(\text{z}\in N\left({\text{z}}_{j},k\right)\right)\right\}, \end{array}$$

where *N*(**z**, *k*) denotes the *k* nearest neighbors of **z**. *R*(**z**, *k*) imposes the condition that **z** and each element of *R*(**z**, *k*) are mutually the *k* nearest neighbors of each other.

**Figure 2:**
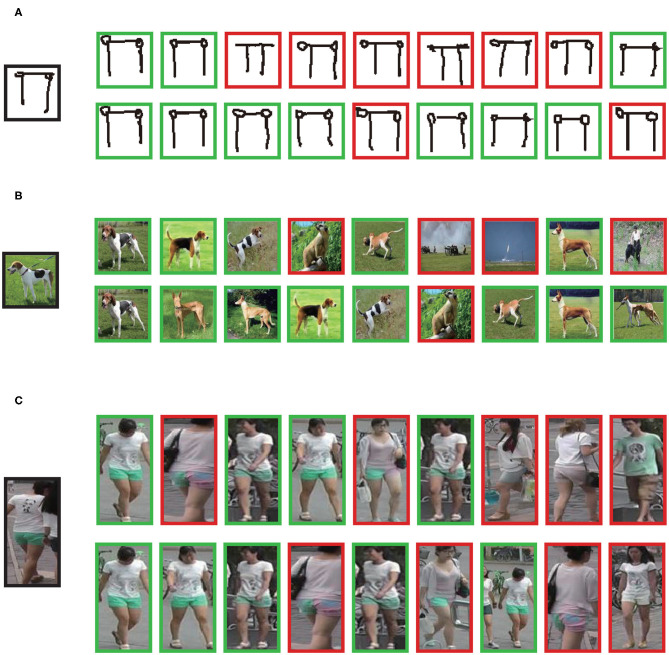
Comparison between k-nearest neighbors and k-reciprocal nearest neighbors. Given an probe (in the black box), nearest neighbors of the example are shown. Examples in green boxes are those in the same class and examples in red boxes are those in different classes. **(A–C)** Examples from Omniglot, MiniImageNet, and FS-Market1501, respectively. The upper row in each panel is the result of k-nearest neighbors and the lower row in each panel is the result of k-reciprocal nearest neighbors. By adopting KRJD, more positive examples (those in the same class) appear in the nearest neighborhood of the probe.

Compared to the Euclidean distance, KRJD takes into account the reciprocal relationship between data points, and hence is a stricter metric measuring whether two feature points match or not. Given a query probe, we find that the results of nearest neighbors based on the KRJD is more accurate than that of the Euclidean distance (i.e., the k-nearest neighbors) as demonstrated in Figure 2 (see Appendix 1 for more detail).

#### 2.2.2. Density-Based Spatial Clustering

To partition feature points and generate pseudo labels, we adopt a clustering method called density-based spatial clustering algorithm (DBSCAN) (Ester et al., 1996). This method regards clusters as the areas of high density separated by low density regions, that is, a cluster is composed of a set of core points (i.e., those points in a high density region close to each other) and a set of non-core points (i.e., those points in the surrounding low density regions close to the core points but not to themselves). Compared to the conventional Kmeans algorithm, DBSCAN has a number of appealing properties: (1) it applies to any shape of clusters, as opposed to the Kmeans algorithm assuming that clusters are convex; (2) it requires no assumption of the number of clusters; (3) it can detect outliers, which is extremely useful for iterative training, as data points are typically intertwined in the first few iterations.

After applying DBSCAN, we get the pseudo label set (the cluster identity), which is expressed as

(3) $$\begin{array}{l} \{y_{i}\}=DBSCAN\left(ms,\epsilon ,\{z_{i}\}\right), \end{array}$$

where the parameter *ms* defines the minimum sample value, i.e., the minimum number of points huddled together for a region to be considered as dense, and the parameter ϵ defines the distance threshold, i.e., the maximum distance for two points to be considered as in the same neighborhood. Higher *ms* or lower ϵ indicate higher density is necessary to form a cluster. Both *ms* and ϵ affect the cluster numbers and the size of clusters. In general, we want the constructed episodic tasks T to be diverse, so that transferable knowledge can be acquired by the few-shot learner. This corresponds to setting small *ms* and ϵ. We will discuss the choice of *ms* and ρ in section 2.5.

### 2.3. Episodic Training

After removing outliers (i.e., those data points in low density regions in the feature space) in DBSCAN, we construct episodic tasks using the remaining pseudo labeled data ${\left\{\left(\tilde{{\text{x}}_{i}},\tilde{y_{i}}\right)\right\}}_{i=1}^{\tilde{N}}$, with $\tilde{N}$ the number of remaining points. For each episodic task *T*_*i*_, we randomly sample *M* classes and *K* + *Q* examples per class as described in section 2.1, with *K* + *Q* ≤ *ms*.

A number of metric loss functions can be used in our model, including the prototypical loss (Snell et al., 2017), the triplet loss (Weinberger and Saul, 2009; Hermans et al., 2017), the contrastive loss (Hadsell et al., 2006), and the center loss (Wen et al., 2016). To save space, here we mainly describe the prototypical loss. More results of using other metric loss functions can be found in Appendix 2. The prototypical loss aims to learn a prototype for each class and then discriminate a novel example based on its distance to all *M* prototypes, which is written as

(4) $$\begin{array}{l} L_{proto}\left(\text{z},{\text{c}}_{p};\theta \right)=\frac{\exp\left(-\|\text{z}-{\text{c}}_{p}{\|}_{2}^{2}\right)}{\overset{M}{\underset{m}{\sum }}\exp\left(-\|\text{z}-{\text{c}}_{m}{\|}_{2}^{2}\right)}, \end{array}$$

where **z** is a data point from the query set of class *p*, and **c**_*m*_ is the prototype of class *m* given by ${\text{c}}_{m}=\underset{{\text{z}}_{i}\in S_{m}}{\sum }\left({\text{z}}_{i}\right)/K$, with *S*_*m*_ the support set of class *m*. In practice, we choose to minimize the negative log value of Equation 4, i.e., $L_{proto}^{\log}\left(\text{z},{\text{c}}_{p};\theta \right)=-\log L_{proto}\left(\text{z},{\text{c}}_{p};\theta \right)$, as the log value better reflects the geometry of the loss function, making it easier to select a suitable learning rate to minimize the loss function.

In summary, the above two steps for data clustering and episodic training are performed iteratively. They facilitate each other, similar to the EM-style algorithm: data clustering frequently generates pseudo labeled data for episodic learning, and the latter improves the feature representations of data, which in return further improve the clustering quality and few-shot learning (see section 4 for more discussions on why the iterative learning works). The pseudo code of UFLST is summarized in Algorithm 1.

**Algorithm 1:** Unsupervised Few-shot Feature Learning via Self-supervised Training (UFLST)


**Input:** Unlabeled data set $X=\left\{{\text{x}}_{i}\right\}$, the few-shot feature embedding $f_{\theta^{0}}$, the training iteration *T*.
**Output:** Trained few-shot embedding $f_{\theta^{T}}$
1: *t* = 0
2: **repeat**
3: **Clustering:**
4: Extracting features {**z**_*i*_} of {**x**_*i*_} using the feature extractor $f_{\theta^{t}}$.
5: Calculating KRJD *J*_*ij*_ based on the K-reciprocal nearest neighbors of any data pairs **z**_*i*_ and **z**_*j*_.
6: Clustering data using DBSCAN and generating pseudo labels {*y*_*i*_}.
7: Removing outliers and obtaining the pseudo labeled data set $\left\{\left(\tilde{{\text{x}}_{i}},\tilde{y_{i}}\right)\right\}$.
8: **Episodic Training:**
9: Constructing a set of episodic tasks $\left\{T_{s}\right\}$; for each task, randomly sampling *M* classes with *K*+*Q* examples per class from $\left\{\left(\tilde{{\text{x}}_{i}},\tilde{y_{i}}\right)\right\}$.
10: Updating model parameters θ^*t*^ by training the few-shot learner on the series of episodic tasks $\left\{T_{s}\right\}$.
11: *t* = *t* + 1
12: **until** *t* = *T*

### 2.4. Datasets

**Omniglot** contains 1,623 different handwritten characters from 50 different alphabets. There are 20 examples per class and each of them was drawn by a different human subject via Amazon's Mechanical Turk. Following Vinyals et al. (2016), we split the data into two parts: 1,200 characters for training and 423 for testing, and we resize the images to 32 × 32, instead of 28 × 28.

**MiniImageNet** is derived from the ILSVRC-12 dataset. We follow the data split as suggested in Ravi and Larochelle (2016), which contains 100 classes including 64 for training, 16 for validating, and 20 for testing. Each class contains 600 colored images of size 84 × 84.

**FS-Market1501** is a person re-identification (Re-ID) dataset modified from the Market1501 dataset (Zheng et al., 2015). The training set contains 12,936 images with 751 pedestrian identities and the testing set contains 16,483 images with the remaining 750 pedestrian identities. All images were resized to 256 × 128. For more details of how to construct FS-Market1501, see Appendix 3.

### 2.5. Implementation Details

When training on Omniglot and MiniImageNet, we set the model architecture to be the same as in the previous works for fair comparison. The model consists of four stacked layers, and each layer comprises 64-filter 3 × 3 convolution, followed by a batch normalization, a ReLU nonlinearity, and 2 × 2 max-pooling. When training on FS-Market1501, due to high variance in pedestrian pose and image illumination, we use Resnet50 pretrained on ImageNet as the backbone, followed by a global max-pooling layer and a batch normalization layer. Omniglot is relatively easy compared to the other two datasets, and therefore we only pre-process data with normalization. For MiniImageNet and FS-Market1501, we randomly flip images horizontally and crop them with random sizes, and then normalize them with the channel-wise mean and standard deviation of the whole dataset. Color information is important to partition images in FS-Market1501 (pedestrians with the same ID vary in pose, view angle, and illumination but not in the color), while it is not that important to partition images in MiniImageNet (Caron et al., 2018). Hence, we discard color information and increase local contrast by adding a linear transformation based on Sobel filters as proposed in Bojanowski and Joulin (2017) and Paulin et al. (2015). For the clustering method DBSCAN, we set *ms* = 2 and ϵ to be the mean of top *P* values of distance pairs, with *P* = ρ*N*(*N* − 1)/2 and ρ = 0.0015. The values of *ms* and ϵ are set to be relatively small to ensure that feature points are well-separated, so that diverse episodic tasks can be constructed (for more details of the choice of *ms* and ϵ, see Appendix 4). For the prototype loss, we used a higher “way” value (*M* = 60) during training, which leads to better performances as empirically observed in Snell et al. (2017). Since it is possible that the numbers of points in some clusters are too small, we only train the model in the M-way 1-shot learning scenario, i.e., *K* = *Q* = 1. The total number of iterations during training is set to be 100, and in each iteration, 500 episodic tasks are constructed. We used Adam with momentum to update model parameters, and the learning rate is set to be 0.001.

## 3. Results

### 3.1. Comparison With Non-episodic Learning Methods

Episodic learning plays a key role in leveraging unsupervised few-shot feature learning. To demonstrate this, we first compare our model with other unsupervised feature learning methods without employing episodic learning. Three such methods are chosen, which are (Denoising) AutoEncoder (Vincent et al., 2008), InfoGAN (Chen et al., 2016), and DeepClustering (Caron et al., 2018) (for the detailed training process of these methods, see Appendix 5). These methods are the typical approaches used to learn useful feature representations, covering a wide range of unsupervised feature learning strategies including reconstruction (prediction), two-player games, discriminative clustering, and so on. For comparison, we use the features extracted by these methods to calculate the prototype of each class directly and perform the M-way K-shot classification. The results are presented in Table 1, which shows that: (1) compared to other unsupervised feature learning methods whose learning objective is different from ours, iterative data clustering and episodic learning improves the few-shot learning performance significantly, even when the Kmeans clustering with the Euclidean distance is used in our model; (2) by applying DBSCAN with the KRJD metric, the performance of our model is improved further to a large extent. Notably, DeepClustering also jointly learns the parameters of a neural network and the cluster assignments of the resulting features. However, it optimizes the feature representations with a relatively simple learning objective (softmax classification) which is not suitable for few-shot classification.

**Table 1:** Performances of our model compared to other non-episodic unsupervised feature learning methods on Omniglot and MiniImageNet.

			**Omniglot**				**MiniImageNet**			
**Methods (M, K)**	**Clustering**	**Metric**	**(5,1)**	**(5,5)**	**(20,1)**	**(20,5)**	**(5,1)**	**(5,5)**	**(5,20)**	**(5,50)**
Baseline	N/A	N/A	57.97	79.25	34.17	59.33	25.91	32.38	37.01	38.95
AutoEncoder	N/A	N/A	53.63	77.34	32.98	55.01	26.17	33.01	37.98	39.39
Denoising autoEncoder	N/A	N/A	59.63	79.89	34.78	60.88	27.81	34.19	39.01	40.11
InfoGAN	N/A	N/A	51.49	76.38	31.01	53.99	29.81	36.47	40.17	42.46
BiGAN+KNN	N/A	N/A	49.55	68.06	27.37	46.70	25.56	31.10	37.31	43.60
BiGAN+LC	N/A	N/A	-	-	-	-	27.08	33.91	44.00	50.41
DeepClustering	Kmeans	Euclidean	59.07	79.81	34.05	60.12	28.91	36.01	39.29	41.98
UFLST	Kmeans	Euclidean	69.54	86.18	47.11	69.19	31.77	43.03	51.35	55.72
UFLST	BSCAN	KRJD	**96.51**	**99.23**	**90.27**	**97.22**	**37.75**	**50.95**	**59.18**	**62.27**

*Baseline performance means training from scratch. Results based on BiGAN are adapted from Hsu et al. (2018). For complete results with confidence intervals, see Appendix 6. The best performances are in bold*.

### 3.2. The Effect of Iterative Training

**Figure 3:**
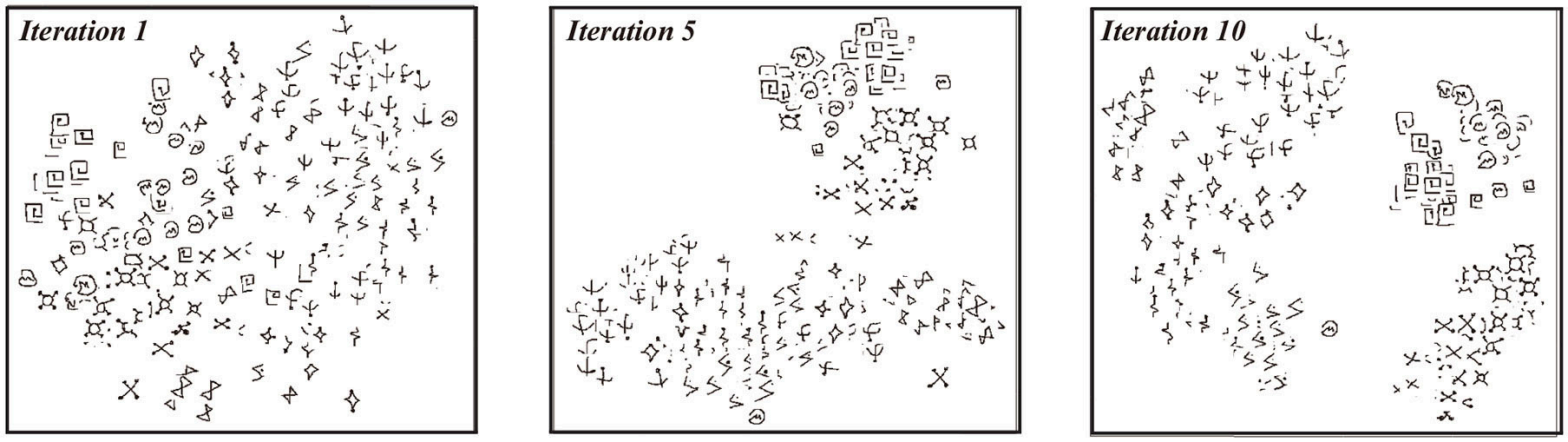
Visualizing clustering results during iterative training with T-SNE. 10 characters from the Futurama alphabets in Omniglot are were selected and results from iteration 1, iteration 5, and iteration 10 are showed here.

**Figure 4:**
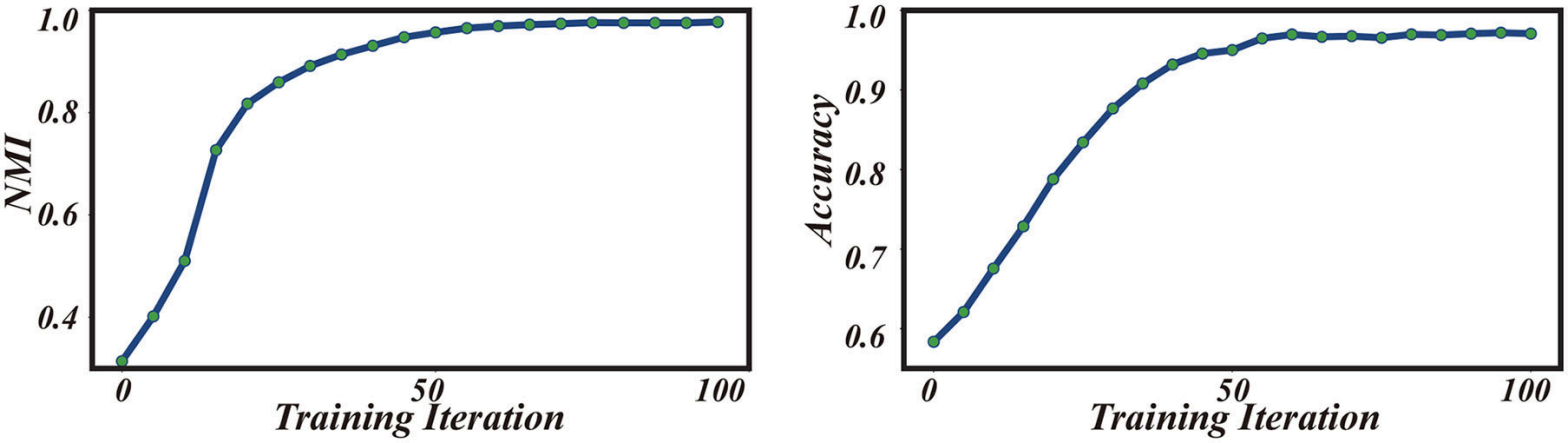
Performances of iterative training under the 5-way 1-shot learning scenario on the Omniglot dataset. **(Left)** NMI vs. training iteration. **(Right)** Classification accuracy vs. training iteration.

In our model, iterative training will gradually improve the clustering quality and the performance of the few-shot learner. To demonstrate this, we randomly select 10 hand-written characters from the Futurama alphabets in Omniglot and visualize clustering behaviors over iteration with T-SNE (Maaten and Hinton, 2008). As shown in Figure 3, initially all data points are intertwined with each other and no clear cluster structure exists. Over training, clusters gradually emerge, in the sense that data points from the same class are grouped together and the margins between different classes are enlarged. This indicates that our model gradually “discovers” the underlying semantic structure of the data. We quantify the clustering quality by computing the normalized Mutual Information (NMI) between the pseudo labels generated by the clustering algorithm $\left\{\tilde{y_{i}}\right\}$ and the ground truth of real labels {ŷ_*i*_}, which is given by,

(5) $$\begin{array}{l} NMI\left(\left\{{ŷ}_{i}\right\},\left\{{\tilde{y}}_{i}\right\}\right)=\frac{I\left(\left\{{ŷ}_{i}\right\},\left\{{\tilde{y}}_{i}\right\}\right)}{\sqrt{H\left(\left\{{ŷ}_{i}\right\}\right)H\left(\left\{{\tilde{y}}_{i}\right\}\right)}}, \end{array}$$

where *I*(·, ·) is the mutual information between {ŷ_*i*_} and $\left\{{\tilde{y}}_{i}\right\}$, and *H*(·) the entropy. The value of NMI lies in [0, 1], with 1 standing for the perfect alignment between two sets. Note that NMI is independent of the permutation of labeling orders. As shown in Figure 4 (left), the value of NMI increases with the training iterations and gradually reaches a high value close to 1. Remarkably, the value of NMI well predicts the classification accuracy of the few-shot learning (Figure 4, right). These results demonstrate that iterative data clustering and episodic training are able to discover the underlying structure of data manifold, and extract the representative features of data necessary for the few-shot classification task.

### 3.3. Comparison With State-of-the-Art Unsupervised Few-Shot Learning Methods

We compare our model with other state-of-the-art unsupervised few-shot learning methods, including CACTUs (Hsu et al., 2018), UMTRA (Khodadadeh et al., 2018), and AAL (Antoniou and Storkey, 2019), as shown in Table 2. On Omniglot, our model outperforms them to a large extent. Remarkably, the best performances of our model approaches that of two supervised methods, which are the upper bounds for unsupervised learning. Our model also achieves significant improvement on MiniImageNet (note that we only test the model under the 5-way few-shot learning scenario). For example, in the 5-way 1-shot scenario, our model achieves 37.75%, which is significant compared to the baseline performance 25.91%.

**Table 2:** Comparison to state-of-the-art unsupervised few-shot learning models on Omniglot and MiniImageNet under different settings.

	**Omniglot**				**MiniImageNet**			
**Methods (M, K)**	**(5,1)**	**(5,5)**	**(20,1)**	**(20,5)**	**(5,1)**	**(5,5)**	**(5,20)**	**(5,50)**
ACAI/DC-CACTUs-MAML (Hsu et al., 2018)	68.84	87.78	48.09	73.36	39.90	**53.97**	**63.84**	**69.64**
ACAI/DC-CACTUs-ProtoNets (Hsu et al., 2018)	68.12	83.58	47.75	66.27	39.18	53.36	61.54	63.55
BiGAN-CACTUs-MAML (Hsu et al., 2018)	58.18	78.66	35.56	58.62	36.24	51.28	61.33	66.91
BiGAN-CACTUs-ProtNets (Hsu et al., 2018)	54.74	71.69	33.40	50.62	36.62	50.16	59.56	63.27
UMTRA+AutoAugment (Khodadadeh et al., 2018)	83.80	95.43	74.25	92.12	**39.93**	50.73	61.11	67.15
AAL-MAML++ (Antoniou and Storkey, 2019)	88.40	97.96	70.21	88.32	33.30	49.18	–	–
AAL-ProtoNets (Antoniou and Storkey, 2019)	84.66	89.14	68.79	74.28	37.67	40.29	–	–
UFLST+Kmeans+Euclidean (ours)	69.54	86.18	47.11	69.19	31.77	43.03	51.35	55.72
UFLST+DBSCAN+KRJD (ours)	**96.51**	**99.23**	**90.27**	**97.22**	37.75	50.95	59.18	62.27
MAML (Finn et al., 2017) (supervised)	98.7	99.9	95.8	98.9	46.81	62.13	71.03	75.54
ProtoNets (Snell et al., 2017) (supervised)	98.8	99.7	96.0	98.9	46.56	62.29	70.05	72.04

*Results based on BiGAN are adapted from Hsu et al. (2018). For complete results with confidence intervals, see Appendix 7. The best performances are in bold*.

We also note that some methods outperform our model on MiniImageNet, e.g., DeepCluster-CACTUs-ProtoNets and UMTRA-AutoAugment achieve 39.18 and 39.93% in the 5-way 1-shot scenario, respectively. The reasons we believe are due to three aspects. Firstly, for the convenience of comparing to other (un)supervised few-shot learning methods, we have used the 4-layer convnet as the few-shot embedding network. Such a simple network is unable to adequately extract the semantic meanings of images under the unsupervised setting, especially as the in-class variations of MiniImageNet are large but the total size of the dataset is small (only 64 classes with 600 images per class in the training set). Secondly, for constructing diverse episodic tasks, our model prefers to over-segment the data into hundreds of clusters, whereas the ground truth cluster number of MiniImageNet is only 64. This induces mismatch between the constructed episodic tasks and the ground truth. Thirdly, the methods outperforming our model adopt either powerful prior unsupervised feature learning to partition data points (the CACTU-based model) or complicated data augmentation strategies to construct the episodic tasks (the UMTRA-based model and the AAL-based model), while our model partitions data points with the features directly extracted from the few-shot embedding network and only adopts a simple data augmentation strategy to avoid overfitting. One solution is to use deeper feature embedders, e.g., Resnet12, AlexNet in our model to improve the performance (see Appendix 9). Even so, our model still achieves competitive results compared to other unsupervised few-shot learning methods.

### 3.4. Results on FS-Market1501

In order to show the applicability of our model to a real-world few-shot learning problem, we apply our model on the FS-Market1501 dataset which has been described in section 2.4. In reality, labeled data is extremely lacking for person Re-ID, and unsupervised learning becomes crucial. Results in Table 3 show that our UFLST model performs very well on the 1-shot learning problem on this dataset. Note that the 1-shot learning problem we demonstrate here is to mimic the typical single query setting in person Re-ID. For example, 50-way 1-shot means the model needs to identify a pedestrian from one of 50 unknown persons by training a classifier with only one image per person. To compare our model with the supervised results as described in section 3.3, we train a supervised model with the same model architecture, i.e., the Resnet50 backbone pretrained on ImageNet as described in section 2.5. Overall, we observe that our model achieves encouraging performances compared to the supervised methods, in particular, in the scenario of low-way classification. This suggests that our model is potentially feasible in practice for person Re-ID when annotated labels are unavailable.

**Table 3:** Performances of our model on FS-Market1501 with different settings.

	**5-way**	**10-way**	**15-way**	**20-way**	**50-way**	**100-way**
Baseline	48.8	35.7	29.7	27.8	20.9	16.4
UFLST-Tripetloss	72.8	63.0	56.2	53.4	42.5	35.4
UFLST-Prototypeloss	88.3	81.2	75.8	73.0	62.5	54.0
UFLST-HardTripletloss	**91.4**	**86.9**	**81.6**	**80.4**	**70.1**	**62.1**
Supervised upper bound	96.8	94.7	92.5	91.1	83.7	77.3

*Only 1-shot learning is considered to mimic the typical single query evaluation condition in person Re-ID. We adopt three metric losses to optimize the model, see Appendix 8 for detail. The best performances are in bold*.

## 4. Conclusion and Discussion

In this study, we have proposed a model UFLST for unsupervised few-shot learning. Different from other unsupervised feature learning methods, such as the prediction-based and the GAN-based ones, our model exploits the paradigm of episodic training, which is a more effective way to implement few-shot learning. Recently, a few unsupervised few-shot learning models based on episodic learning were proposed, and they have taken different strategies to construct episodic tasks from unlabeled data. For instance, CACTUs constructs episodic tasks by partitioning the features extracted by a prior-trained unsupervised feature embedding network with different objective functions and then train the few-shot learner (Hsu et al., 2018). UMTRA utilizes a domain-specific data augmentation strategy to generate synthetic tasks for the meta-learning phase, while in such a way, the constructed episodic tasks are restricted by the data augmentation strategy (Khodadadeh et al., 2018). Different from the above methods, we propose a simple yet effective way to construct episodic tasks, that is, we partition the features directly from the few-shot embedding network and do this in an iterative manner along with the training of the few-shot learner; and by this, the construction of episodic tasks and the training of few-shot learner are improved concurrently. Furthermore, to improve the clustering quality, we have proposed to use the k-reciprocal Jaccard distance metric to reduce false positive examples during the clustering.

We have demonstrated encouraging performances of our model on two benchmark datasets, Omniglot, and MiniImageNet. We also constructed a new dataset called FS-Market1501 adapted from Market1501 to test our model, and demonstrated the feasibility of our model to real-world applications. The high efficiency of our model also prompts us to think about why it works. The key of our model is the iterative implementation of data clustering and episodic training, and they tend to facilitate each other as the EM-style algorithm. At the beginning of training, the few-shot embedding network is randomly initialized, and the embedded features are intertwined with each other, making the constructed episodic tasks very noisy. However, even in such a situation, the embedded features are not completely random as observed in Noroozi and Favaro (2016), which showed that the performance of a randomly initialized convnet is above the chance level. For example, a simple multilayer perceptron built on top of the last convolutional layer of a random AlexNet achieves 12% accuracy on ImageNet, while the chance level is only 0.1%. This implies that this weak signal can be exploited to bootstrap the discriminative power of our model through iterative training. As shown in Figures 3, 4, data clustering and feature extraction in our model facilitate each other, which eventually produces a well-performed few-shot learner. To our knowledge, our work is the first one that integrates progressive clustering and episodic training for unsupervised few-shot learning. Notably, the idea of unsupervised iterative learning of our model agrees with the self-learning nature of humans. It will be interesting to further explore the relationship between human learning and machine learning on unsupervised few-shot learning.

## Data Availability Statement

All datasets generated for this study are included in the article/Supplementary Material.

## Author Contributions

ZJ designed the study, performed the experiments, and wrote the first draft of the manuscript. XZ helped with integrating algorithms and conducting experiments. TH and SW contributed to the conception and design of the study and revision. ZJ and SW wrote the final manuscript. All authors contributed to the article and approved the submitted version.

## Conflict of Interest

The authors declare that this study received funding from Huawei Technology Co., Ltd (YBN2019105137). The funder was not involved in the study design, collection, analysis, interpretation of data, the writing of this article or the decision to submit it for publication.
