# Supplementary material for: Unsupervised Few-Shot Feature Learning via Self-Supervised Training
Source: Front Comput Neurosci. 2020 Oct 14;14:83. doi: 10.3389/fncom.2020.00083 (PMC7592391; doi:10.3389/fncom.2020.00083)
Supplement: Supplementary file 1 [file Table_1.pdf]

Supplementary Material

1 COMPARISON BETWEEN K-NEAREST NEIGHBOURS AND K-RECIPROCAL NEAREST NEIGHBOURS

Here, we present more examples from three datasets by using the KRJD metric during clustering. Given an probe (in the black box), nearest neighbours of the probe are shown. Examples in green boxes are those of the same class as the probe and examples in red boxes are those of different classes. Fig. S1, Fig. S2 and Fig. S3 are examples from Omniglot, MiniImageNet and FS-Market1501, respectively. The upper row in each panel is the result of k-nearest neighbours and the lower row is the result of k-reciprocal nearest neighbours. By adopting KRJD, more positive examples (those in the same class) appear in the nearest neighbourhood of the probe, demonstrating that the KRJD metric is effective to boost the performance of our model.

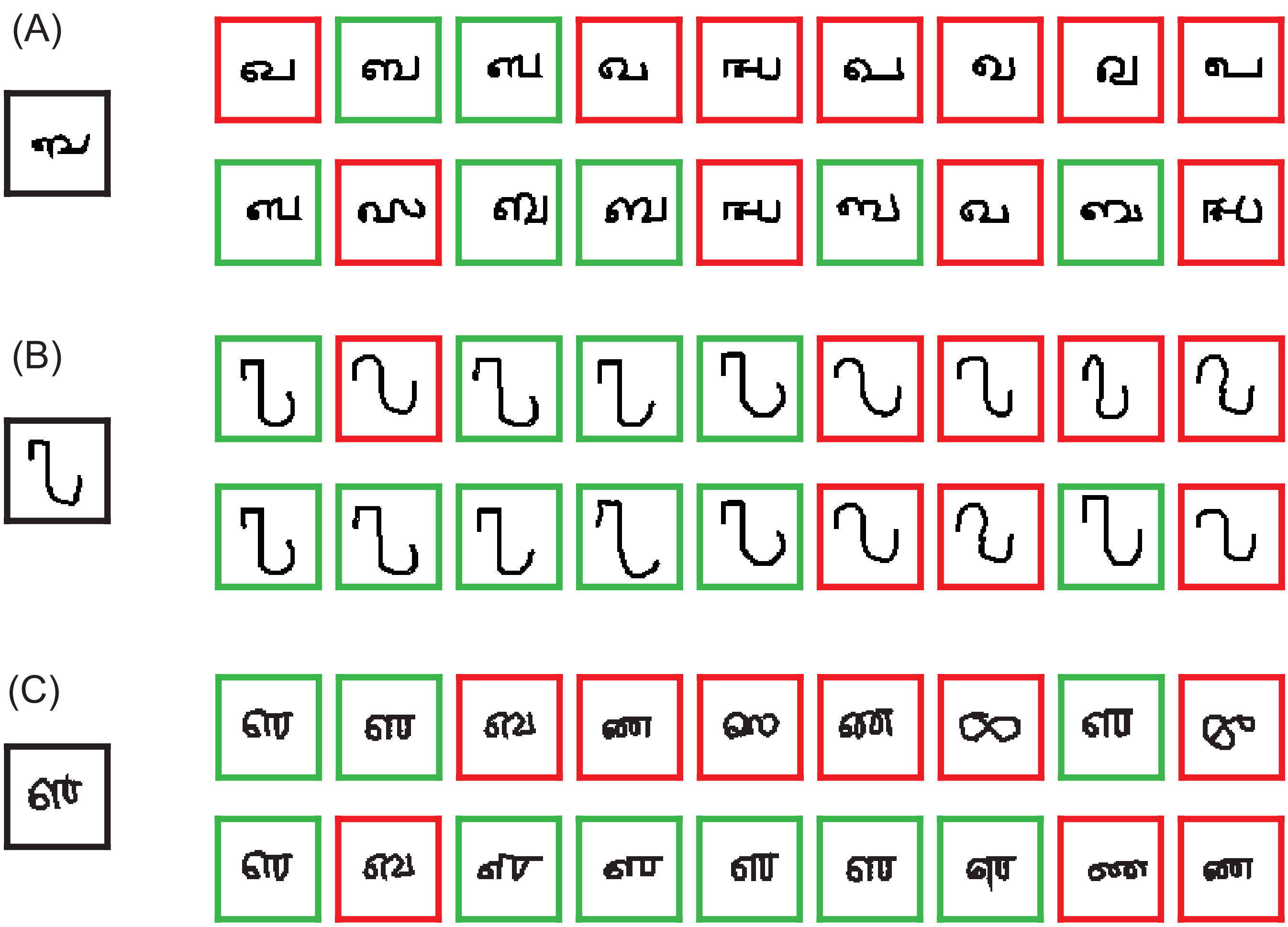

Figure S1. Comparison between k-nearest neighbours and k-reciprocal nearest neighbours on Omniglot dataset.

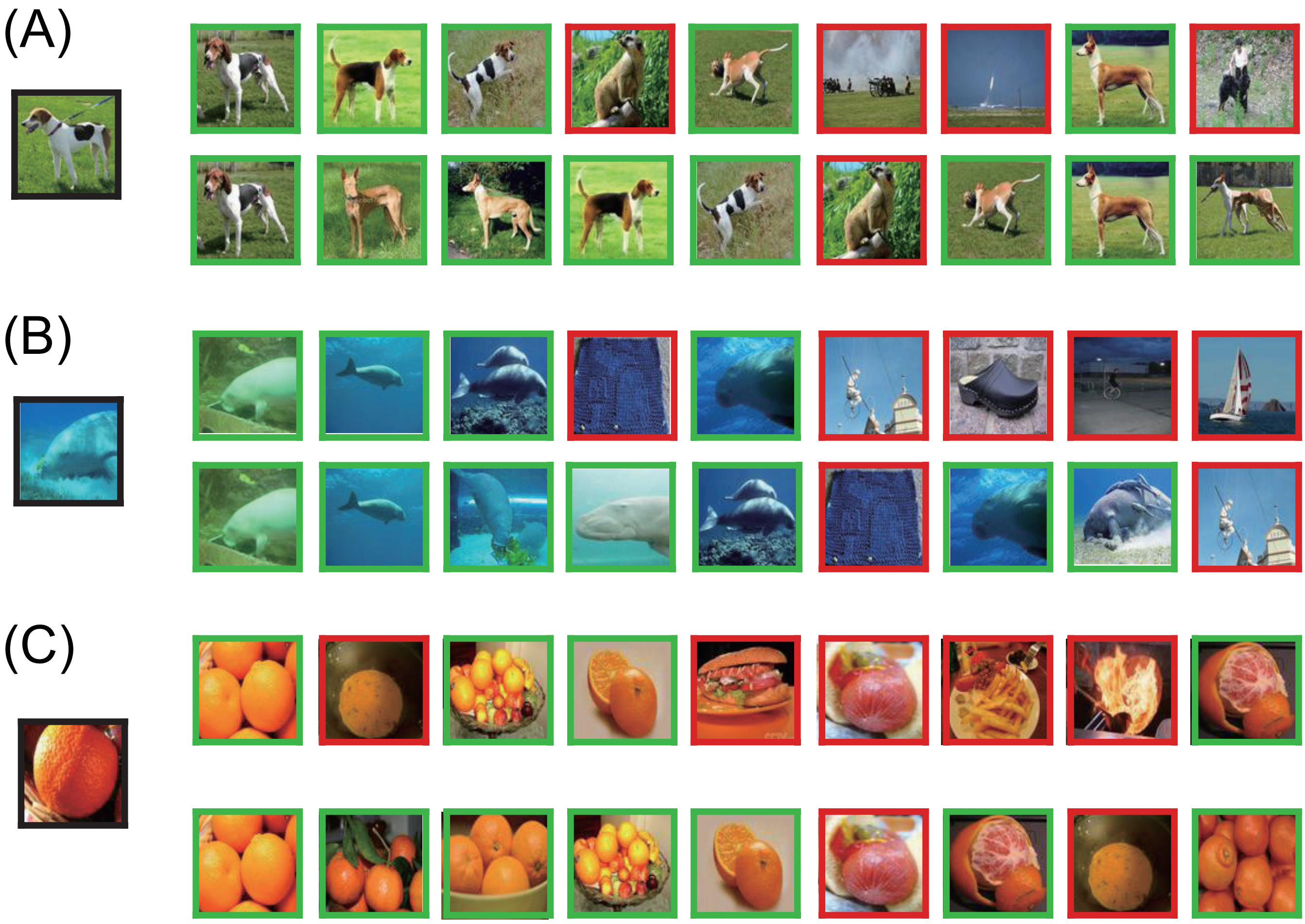

Figure S2. Comparison between k-nearest neighbours and k-reciprocal nearest neighbours on MiniImageNet dataset.

2 OTHER METRIC LOSS FUNCTIONS USED IN OUR MODEL

The goal of our study is to find a good embedding feature space from the unlabeled dataset $\mathcal{X} : \{\mathbf{x}_i\}$, so that we can build a few-shot classifier which can be directly applied on the downstream tasks efficiently. Theoretically, many metric loss functions can be used in our model. Here we present the results on the triplet loss (Weinberger and Saul, 2009) and the hardtriplet loss (Hermans et al., 2017) (Table S1, Table S2). They have been widely used in face recognition and image retrieval. The triplet loss $L_{triplet}$ consists of several triplets, each of which includes a query feature $\mathbf{z}$, a positive feature $\mathbf{z}^+$ and a negative feature $\mathbf{z}^-$, and is written as

$$L_{triplet}(\mathbf{z}, \mathbf{z}^+, \mathbf{z}^-; \theta) = \max(0, \|\mathbf{z} - \mathbf{z}^+\|_2^2 - \|\mathbf{z} - \mathbf{z}^-\|_2^2 + m), \tag{S1}$$

where m controls the margin of two classes, and the hinge term plays the role of correcting triplets, so that the difference between the similarities of positive and negative examples to the query point is larger than a margin m. However, in the above form, positive pairs in those "already correct" triplets will no longer be pulled together due to the hard cutoff. We therefore replace the hinge term by a soft-margin formulation,

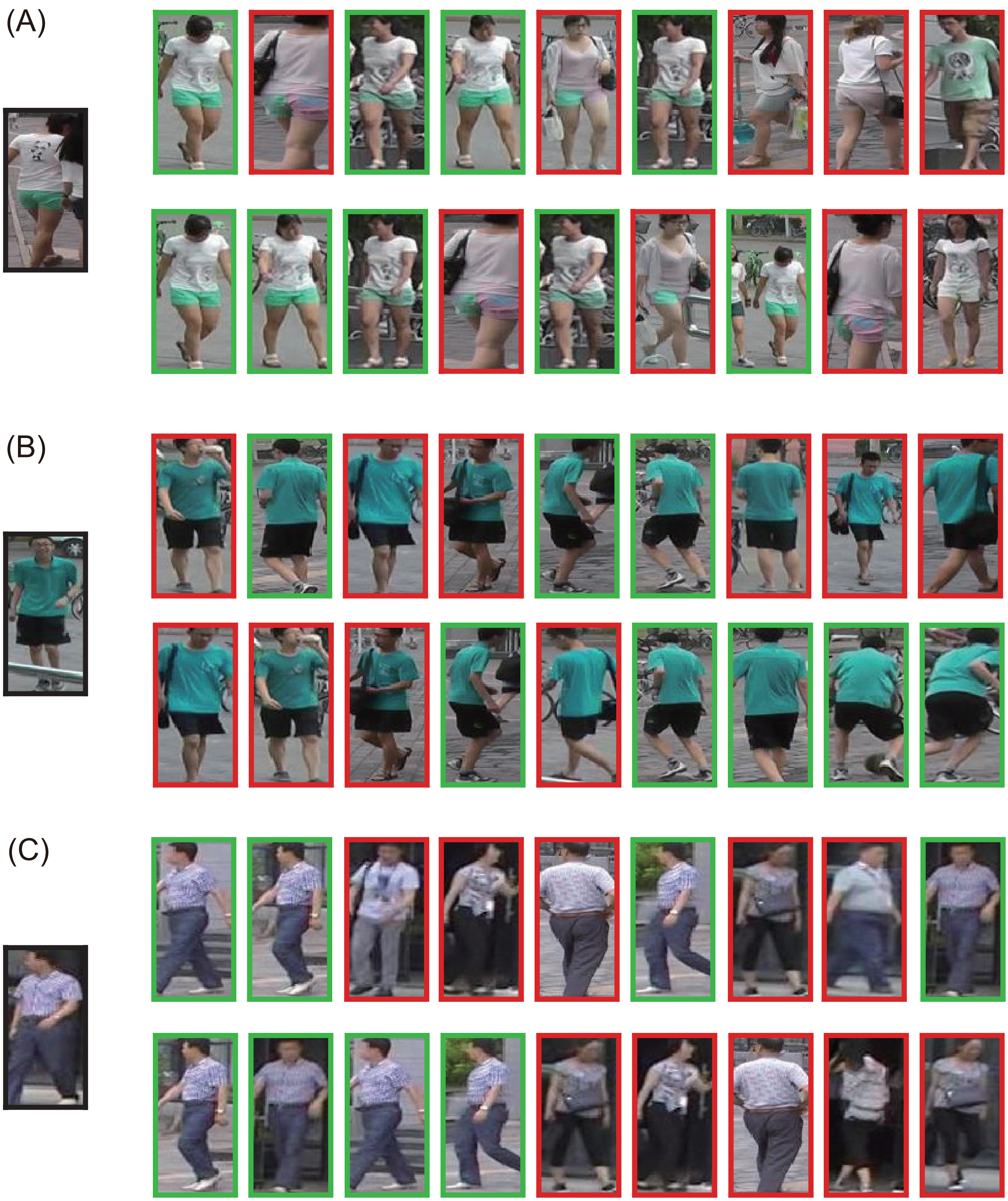

Figure S3. Comparison between k-nearest neighbours and k-reciprocal nearest neighbours on FS-Market1501 dataset.

which gives

$$L_{triplet-SM}(\mathbf{z}, \mathbf{z}^+, \mathbf{z}^-; \theta) = \log\left[1 + \exp(\|\mathbf{z} - \mathbf{z}^+\|_2^2 - \|\mathbf{z} - \mathbf{z}^-\|_2^2 + m)\right]. \tag{S2}$$

Eq. S2 is similar to Eq. S1, but it decays exponentially instead of having a hard cutoff and tends to be numerically more stable (Hermans et al., 2017).

Here we simply discuss the relationships between the triplet loss and the prototypical loss we used in the main text. Consider a M-way 1-shot episodic learning scenario, where a prototype $\mathbf{c_k}$ is the support point $\mathbf{z_k}$ itself, the prototypical loss (Equ. 4 in the main text) is written as

$$\begin{aligned} L^{\log}_{proto}(\mathbf{z}, \mathbf{z}_k; \theta) &= -\log \frac{\exp(-\|\mathbf{z} - \mathbf{z_p}\|_2^2)}{\sum_k \exp(-\|\mathbf{z} - \mathbf{z}_k\|_2^2)}, \\ &= -\log \frac{1}{1 + \sum_{k \neq p} \exp(\|\mathbf{z} - \mathbf{z}_p\|_2^2 - \|\mathbf{z} - \mathbf{z}_k\|_2^2)}, \\ &= \log\left[1 + \sum_{k \neq p} \exp(\|\mathbf{z} - \mathbf{z}_p\|_2^2 - \|\mathbf{z} - \mathbf{z}_k\|_2^2)\right]. \end{aligned} \tag{S3}$$

From Eq. S3, we can see that query point $\mathbf{z}$ is pulled towards the corresponded support point $\mathbf{z}_p$, and meanwhile, $\mathbf{z}$ is pushed away from all other support points $\{\mathbf{z}_k\}_{k \neq p}$; whereas, when using the triplet soft-margin loss (Eq. S2), the query point $\mathbf{z}$ is only pushed away from one negative points $\mathbf{z}^-$. This implies that in each update, $L_{triplet-SM}$ only interacts with a single negative example from one of other classes and ignores many other negative examples. When K is small, optimizing the model with the two loss functions has no big difference. For example, when $K = 2$ and $m = 0$, Eq. S2 and S3 become exactly the same. However, when K becomes larger, the possible number of triplets grows cubically with M and linearly with K, which makes it difficult to select non-trivial triplets. In such a situation, optimizing on these uninformative triplets leads to the problem that the model gets stuck into a local optimum and suffers slow convergence. This justifies why the model has a inferior performance using the triplet loss compared to using the prototype loss (Table S1 and Table S2).

The inefficiency of the conventional triplet loss motivate us to mine hard triplets to alleviate its shortcomings (Wang et al., 2014; Cui et al., 2016; Hermans et al., 2017). Mining hard negative examples across the whole dataset is infeasible, since it is too time-costing to evaluate all embedding vectors in the deep learning framework. So, we choose to do hard negative example mining within a batch, i.e., we select the hardest positive and the hardest negative examples when forming the triplets, and obtain

$$L^{hard}_{triplet-SM} = \log\left[1 + \exp(\max_{\mathbf{z}_p \in \{\mathbf{z}^+\}} \|\mathbf{z} - \mathbf{z_p}\|_2^2 - \min_{\mathbf{z}_n \in \{\mathbf{z}^-\}} \|\mathbf{z} - \mathbf{z}_n\|_2^2 + m)\right]. \tag{S4}$$

Compared to Eq. S3 which pushes a query point away from all other support points from different classes, Eq. S4 focuses on pulling the hardest positive example closer and pushing the hardest negative example away at the same time. By this, we get a slighbetter performance than that using the prototype loss (Table S1, Table S2).

	5-way Acc.		20-way Acc.	
	1-shot	5-shot	1-shot	5-shot
Tripletloss	88.68	96.65	73.21	90.11
Prototypeloss	96.51	**99.23**	90.27	97.22
HardTripletloss	**97.03**	99.19	**91.28**	**97.37**

Table S1. Different metric loss functions used in our model on Omniglot.

	5-way	10-way	15-way	20-way	50-way	100-way
Tripetloss	72.8	63.0	56.2	53.4	42.5	35.4
Prototypeloss	88.3	81.2	75.8	73.0	62.5	54.0
HardTripletloss	**91.4**	**86.9**	**81.6**	**80.4**	**70.1**	**62.1**

Table S2. Different metric loss functions used in our model on FS-Market1501. Only 1-shot learning is considered to mimic the typical single query condition in person Re-ID applications.

	5-way Acc.		20-way Acc.	
	1-shot	5-shot	1-shot	5-shot
$\rho = 0.0001$	78.88	92.73	59.49	81.50
$\rho = 0.0002$	82.85	94.82	64.83	85.61
$\rho = 0.0003$	91.43	97.79	78.48	93.04
$\rho = 0.0004$	97.57	**99.35**	92.62	97.82
$\rho = 0.0005$	97.03	99.19	91.28	97.37
$\rho = 0.0006$	**97.64**	99.34	**93.39**	**98.06**
$\rho = 0.0007$	97.16	99.12	91.28	97.48
$\rho = 0.0008$	97.49	99.25	92.14	97.72

Table S3. Effects of the ρ value in DBSCAN on the Omniglot dataset.

Dataset	Market1501	FS-Market1501
training identities	751	751
training images	12936	12936
testing identities	750	750
testing images	19732	16483
distractors	2793	0
identity “0”	456	0

Table S4. Comparison between Market1501 dataset and FS-Market1501 dataset.

3 CONSTRUCTION OF THE FS-MARKET1501 DATASET

FS-Market1501 is a person re-identification (Re-ID) dataset constructed from the Market1501 dataset. In the original dataset, a total of six cameras were used, including 5 high-resolution cameras, and one low-resolution camera to collect images. Overall, the original dataset contains 32,668 annotated bounding boxes of 1,501 identities, including 12936 images with 751 pedestrian identities for training, 3368 images with 750 pedestrian identities for query and the remaining images as the gallery set. To improve the retrieval difficulty, the original gallery set also contains some distractors, e.g., the low DPM value images and the images of identity “0”. When constructing the FS-Market1501 dataset, we remove the distrators from the gallery set and keep the remaining as well as the query set as our testing set. Totally, there are 12936 images with 751 pedestrian identities for training and 16483 images with the remaining 750 pedestrian identities for evaluating the few-shot performance of our model(see Table S4).

Methods (M, K)	Clustering	Metric	Omniglot (5,1)	(5,5)	(20,1)	(20,5)
Baseline	N/A	N/A	57.97±0.84	79.25±0.67	34.17±0.45	59.33±0.41
AutoEncoder	N/A	N/A	53.63±0.99	77.34±0.65	32.98±0.43	55.01±0.33
Denoising AutoEncoder	N/A	N/A	59.63±0.88	79.89±0.59	34.78±0.44	60.88±0.41
InfoGAN	N/A	N/A	51.49±0.97	76.38±0.71	31.01±0.60	53.99±0.49
BiGAN+KNN	N/A	N/A	49.55±1.27	68.06±0.71	27.37±0.33	46.70±0.36
DeepClustering	Kmeans	Euclidean	59.07±0.91	79.81±0.76	34.05±0.51	60.12±0.48
UFLST (Ours)	Kmeans	Euclidean	69.54±0.78	86.18±0.64	47.11±0.49	69.19±0.41
UFLST (Ours)	BSCAN	KRJD	**96.51±0.88**	**99.23±0.67**	**90.27±0.48**	**97.22±0.49**

Table S5. Peformances of our model compared to other non-episodic unsupervised feature learning methods on Omniglot. All accuracy results are averaged over 1000 test episodes and are reported with 95% confidence intervals.

Methods (M, K)	Clustering	Metric	MiniImageNet (5,1)	(5,5)	(5,20)	(5,50)
Baseline	N/A	N/A	25.91±1.01	32.38±0.91	37.01±0.76	38.95±0.84
AutoEncoder	N/A	N/A	26.17±0.91	33.01±0.81	37.98±0.70	39.39±0.73
Denoising AutoEncoder	N/A	N/A	27.81±0.78	34.19±0.71	39.01±0.67	40.11±0.61
InfoGAN	N/A	N/A	29.81±0.81	36.47±0.71	40.17±0.72	42.46±0.62
BiGAN+KNN	N/A	N/A	25.56±1.08	31.10±0.63	37.31±0.40	43.60±0.37
BiGAN+LC	N/A	N/A	27.08±1.24	33.91±0.64	44.00±0.45	50.41±0.37
DeepClustering	Kmeans	Euclidean	28.91±0.89	36.01±0.71	39.29±0.81	41.98±0.56
UFLST (Ours)	Kmeans	Euclidean	31.77±0.75	43.03±0.71	51.35±0.69	55.72±0.61
UFLST(Ours)	DBSCAN	KRJD	**37.75±0.78**	**50.95±0.71**	**59.18±0.72**	**62.27±0.67**

Table S6. Peformances of our model compared to other non-episodic unsupervised feature learning methods on MiniImageNet. All accuracy results are averaged over 1000 test episodes and are reported with 95% confidence intervals.

Methods (M, K)	Omniglot (5,1)	(5,5)	(20,1)	(20,5)
ACAI/DC-CACTUs-MAML (Hsu et al., 2018)	68.84±0.80	87.78±0.5	48.09±0.41	73.36±0.34
ACAI/DC-CACTUs-ProtoNets (Hsu et al., 2018)	68.12±0.84	83.58±0.61	47.75±0.43	66.27±0.37
BiGAN-CACTUs-MAML (Hsu et al., 2018)	58.18±0.81	78.66±0.65	35.56±0.36	58.62±0.38
BiGAN-CACTUs-ProtNets (Hsu et al., 2018)	54.74±0.82	71.69±0.73	33.40±0.37	50.62±0.39
UMTRA+AutoAug (Khodadadeh et al., 2018)	83.80	95.43	74.25	92.12
AAL-MAML++ (Antoniou and Storkey, 2019)	88.40±0.75	97.96±0.32	70.21±0.86	88.32±1.22
AAL-ProtoNets (Antoniou and Storkey, 2019)	84.66±0.70	89.14±0.27	68.79±1.03	74.28±0.46
UFLST + Kmeans + Euclidean (Ours)	69.54±0.78	86.18±0.64	47.11±0.49	69.19±0.41
UFLST + DBSCAN + KRJD (Ours)	**96.51±0.88**	**99.23±0.67**	**90.27±0.48**	**97.22±0.49**
MAML Finn et al. (2017) (Supervised)	94.46±0.35	98.83±0.12	84.60±0.32	96.29±0.13
ProtoNets Snell et al. (2017) (Supervised)	98.35±0.22	99.58±0.09	95.31±0.18	98.81±0.07

Table S7. Comparison to state-of-the-art unsupervised few-shot learning models on Omniglot under different settings. All accuracy results are averaged over 1000 test episodes and are reported with 95% confidence intervals. The supervised results are borrowed from Hsu et al. (2018).

4 THE CHOICE OF MS AND ρ IN THE DBSCAN ALGORITHM

In the main text, we have demonstrated that for the clustering method DBSCAN, we set $ms = 2$ and ϵ to be the mean of top P values of distance pairs, with $P = \rho N(N-1)/2$ and $\rho = 0.0015$. These values are set to be relatively small to ensure that feature points are well separated, so that diverse episodic tasks can be constructed. Here we analysis the effect of varying ρ when ms is fixed on the Omniglot dataset (see Table S3). The effect of varying ms when fixing ρ is the same.

Methods (M, K)	MiniImageNet (5,1)	(5,5)	(5,20)	(5,50)
ACAI/DC-CACTUs-MAML (Hsu et al., 2018)	39.90±0.74	**53.97±0.70**	**63.84±0.70**	**69.64±0.63**
ACAI/DC-CACTUs-ProtoNets (Hsu et al., 2018)	39.18±0.71	53.36±0.70	61.54±0.68	63.55±0.64
BiGAN-CACTUs-MAML (Hsu et al., 2018)	36.24±0.74	51.28±0.68	61.33±0.67	66.91±0.68
BiGAN-CACTUs-ProtNets (Hsu et al., 2018)	36.62±0.70	50.16±0.73	59.56±0.68	63.27±0.67
UMTRA+AutoAug (Khodadadeh et al., 2018)	**39.93**	50.73	61.11	67.15
AAL-MAML++ (Antoniou and Storkey, 2019)	33.30±0.31	49.18±0.47	-	-
AAL-ProtoNets (Antoniou and Storkey, 2019)	37.67±0.39	40.29±0.68	-	-
UFLST + Kmeans + Euclidean (Ours)	31.77±0.75	43.03±0.71	51.35±0.69	55.72±0.61
UFLST + DBSCAN + KRJD (Ours)	37.75±0.78	50.95±0.71	59.18±0.72	62.27±0.67
MAML (Finn et al., 2017) (Supervised)	46.81±0.77	62.13±0.72	71.03±0.69	75.54±0.62
ProtoNets (Snell et al., 2017) (Supervised)	46.56±0.76	62.29±0.71	70.05±0.65	72.04±0.60

Table S8. Comparison to state-of-the-art unsupervised few-shot learning models on MiniImageNet under different settings. All accuracy results are averaged over 1000 test episodes and are reported with 95% confidence intervals.

Methods (M, K)	MiniImageNet (5,1)	(5,5)	(5,20)	(5,50)
UFLST with 4-layer Convs	37.75±0.78	50.95±0.71	59.18±0.72	62.27±0.67
UFLST with AlexNet	38.10±0.79	51.41±0.68	60.10±0.62	63.45±0.69
UFLST with Resnet12	**39.75±0.73**	**53.95±0.75**	**62.18±0.67**	**68.12±0.61**

Table S9. The model performance on MiniImageNet with deeper embedding networks.

5 TRAINING DETAILS OF THE UNSUPERVISED FEATURE LEARNING METHODS: AUTOENCODER, INFOGAN AND DEEPCLUSTERING

In Sec.4.3 (main text), we compared our model with some unsupervised feature learning methods: (Denoising) AutoEncoder (Vincent et al., 2008), InfoGAN (Chen et al., 2016), and DeepClustering (Caron et al., 2018). For a fair comparison, we modified the feature extractor (the encoder in the AutoEncoder model, the discriminator in the InfoGAN and the feature embedding network in the DeepClustering) to be the 4-layer network as described in Sec.4.2 (main text).

AutoEncoder: we both run AutoEncoder and Denoising AutoEncoder in the current study. We don't use the form of parameter sharing, that is, the decoder has weights that are the transpose of the encoder weights. The model is trained for 200 epochs in total. We used Adam with momentum to update parameters in the encoder and the decoder, and the learning rate is set to 0.005 with an exponential decay after 100 epochs. The mini-batch size is 128.

InfoGAN: the model is an information-theoretic extension to the Generative Adversarial Network that is able to learn disentangled representations in a completely unsupervised manner. When training, we build upon the code which can be found at `https://github.com/Natsu6767/InfoGAN-PyTorch`. On the omniglot dataset, we set the dimension of incompressible noise to be 26, a categorical code with dimension 10, and two continuous codes that can capture variations that are continuous in nature. On the MiniImageNet dataset, we set the dimension of incompressible noise to be 128, a categorical code with dimension 10, and 10 continuous codes.

DeepClustering: the model jointly learns the parameters of a neural network and the cluster assignments of the resulting features. The main contribution of their work is to solve the degenerated solution problem in progressive clustering by reassigning empty clusters during the Kmeans optimization. We follow the training

details in the authors' paper and train a 4-layer feature embedding network with a softmax classification learning objective. The number of clusters is set too be 1000 in both Omniglot and MiniImageNet. The readout layer is re-initialized after Kmeans clustering in each iteration. The number of iterations is set to be 20 and the training epochs in each iteration is set to be 50. The initial learning rate in each iteration is 0.005 with an exponential decay at epoch 25. The mini-batch size is 128.

6 PERFORMANCES OF OUR MODEL COMPARED TO OTHER NON-EPISODIC UNSUPERVISED FEATURE LEARNING METHODS WITH CONFIDENCE INTERVALS

After obtaining the feature extractor in three unsupervised feature learning models, we simply build a prototypical classifier to perform few-shot classification on downstream tasks, that is, performing classification by computing distances to prototype representations of each class. Other methods can be also used to perform few-shot classification on top of the embedding network, such as the K-nearest neighbour, the linear classifier and the multi-layer perceptron. These methods don't benefit from the episodic learning paradigm and cause the probelm of meta-overfitting, as reported in Hsu et al. (2018). Hence, we only run a prototypical classifier on top of these feature embedding networks in the current study (see Table S5 and Table S6).

7 PEFORMANCES OF OUR MODEL COMPARED TO THE SOTA UNSUPERVISED FEW-SHOT LEARNING MODELS WITH CONFIDENCE INTERVALS

Note that there is no confidence intervals reported in the UMTRA model. The confidence intervals on the supervised learning methods MAML and ProtoNets are borrowed from Hsu et.al hsu2018unsupervised (see Table S7 and Table S8).

8 SUPERVISED TRAINING ON THE FS-MARKET1501

Resnet50 pretrained on Imagenet is a conventional backbone model on person ReID benchmarks. In the current study, we also use it as our backbone model on the FS-Market1501 dataset. Following (Xiong et al., 2018), we add a batch normalization layer after the global pooling layer to prevent overfitting and directly use the batch-normalized global pooling features to calculate the prototype of each class. When training with triplet loss and hardtriplet loss, the margin m between negative pairs and positive pairs is set as 0.3. When training with prototype loss, the setting is the same as decribed in Sec.4.2. For the results, see Table S2.

9 USING RESNET12 AND ALEXNET AS THE FEATURE EMBEDDING NETWORK ON MINIIMAGENET

In Sec.4.4, we showed that the performance of our model on MiniImageNet is competitive to other SOTA unsupervised few-shot learning methods, but not one of the SOTA models. One possible reason is that the feature embedding network is too simple (a 4-layer convnet) to extract the semantic meaning of images, especially under the unsupervised setting. In other words, a shallow embedding network did not make adequate use of UFLST's expressive capacity, and opted to use a deeper embedding network to prevent underfitting. Here we use Resnet12 and AlexNet as the feature embedding network which are more complex that the 4-layer convnet to improve the performance of unsupervised few-shot learning. The Resnet12 has been used in several supervised few-shot learning models (Mishra et al., 2017; Oreshkin et al., 2018), which is a smaller version of Resnet (He et al., 2016). The AlexNet is proposed by Krizhevsky et al. (2012) which has had a large impact on the field of machine learning. Table S9 shows that when using a deeper

embedding network, the few-shot classification performance on MiniImageNet is improved compared to the shallow embedding network used in our model. Our model achieves 38.10%, 39.75% under the 5-way 1-shot scenario with AlexNet and Resnet12, respectively, which is the state-of-the-art results under the unsupervised few-shot learning paradigm on MiniImageNet.

REFERENCES

Antoniou, A. and Storkey, A. (2019). Assume, augment and learn: Unsupervised few-shot meta-learning via random labels and data augmentation. *arXiv preprint arXiv:1902.09884*

Caron, M., Bojanowski, P., Joulin, A., and Douze, M. (2018). Deep clustering for unsupervised learning of visual features. In *Proceedings of the European Conference on Computer Vision (ECCV)*. 132–149

Chen, X., Duan, Y., Houthooft, R., Schulman, J., Sutskever, I., and Abbeel, P. (2016). Infogan: Interpretable representation learning by information maximizing generative adversarial nets. In *Advances in neural information processing systems*. 2172–2180

Cui, Y., Zhou, F., Lin, Y., and Belongie, S. (2016). Fine-grained categorization and dataset bootstrapping using deep metric learning with humans in the loop. In *Proceedings of the IEEE conference on computer vision and pattern recognition*. 1153–1162

Finn, C., Abbeel, P., and Levine, S. (2017). Model-agnostic meta-learning for fast adaptation of deep networks. In *Proceedings of the 34th International Conference on Machine Learning-Volume 70* (JMLR. org), 1126–1135

He, K., Zhang, X., Ren, S., and Sun, J. (2016). Deep residual learning for image recognition. In *Proceedings of the IEEE conference on computer vision and pattern recognition*. 770–778

Hermans, A., Beyer, L., and Leibe, B. (2017). In defense of the triplet loss for person re-identification. *arXiv preprint arXiv:1703.07737*

Hsu, K., Levine, S., and Finn, C. (2018). Unsupervised learning via meta-learning. *arXiv preprint arXiv:1810.02334*

Khodadadeh, S., Bölöni, L., and Shah, M. (2018). Unsupervised meta-learning for few-shot image and video classification. *arXiv preprint arXiv:1811.11819*

Krizhevsky, A., Sutskever, I., and Hinton, G. E. (2012). Imagenet classification with deep convolutional neural networks. In *Advances in neural information processing systems*. 1097–1105

Mishra, N., Rohaninejad, M., Chen, X., and Abbeel, P. (2017). A simple neural attentive meta-learner. *arXiv preprint arXiv:1707.03141*

Oreshkin, B., López, P. R., and Lacoste, A. (2018). Tadam: Task dependent adaptive metric for improved few-shot learning. In *Advances in Neural Information Processing Systems*. 721–731

Snell, J., Swersky, K., and Zemel, R. (2017). Prototypical networks for few-shot learning. In *Advances in Neural Information Processing Systems*. 4077–4087

Vincent, P., Larochelle, H., Bengio, Y., and Manzagol, P.-A. (2008). Extracting and composing robust features with denoising autoencoders. In *Proceedings of the 25th international conference on Machine learning*. 1096–1103

Wang, J., Song, Y., Leung, T., Rosenberg, C., Wang, J., Philbin, J., et al. (2014). Learning fine-grained image similarity with deep ranking. In *Proceedings of the IEEE Conference on Computer Vision and Pattern Recognition*. 1386–1393

Weinberger, K. Q. and Saul, L. K. (2009). Distance metric learning for large margin nearest neighbor classification. *Journal of Machine Learning Research* 10, 207–244

Xiong, F., Xiao, Y., Cao, Z., Gong, K., Fang, Z., and Zhou, J. T. (2018). Towards good practices on building effective cnn baseline model for person re-identification. *arXiv preprint arXiv:1807.11042*
